# Exploring a novel method for optimising the implementation of a colorectal cancer risk prediction tool into primary care: a qualitative study

**DOI:** 10.1186/s13012-022-01205-8

**Published:** 2022-05-12

**Authors:** Shakira Milton, Jon D. Emery, Jane Rinaldi, Joanne Kinder, Adrian Bickerstaffe, Sibel Saya, Mark A. Jenkins, Jennifer McIntosh

**Affiliations:** 1 Centre for Cancer Research, University of Melbourne, Melbourne, Australia; 2 Department of General Practice, University of Melbourne, Melbourne, Australia; 3 The Primary Care Unit, Institute of Public Health, University of Cambridge School of Clinical Medicine, Box 113, Cambridge Biomedical Campus, Cambridge, CB2 0SR UK; 4 University of Melbourne Shepparton Medical Centre, Melbourne Teaching Health Clinics Ltd, 49 Graham Street, Shepparton, VIC 3630 Australia; 5 Centre for Epidemiology and Biostatistics, Melbourne School of Population and Global Health, University of Melbourne, Parkville, Victoria Australia; 6 HumaniSE Lab, Department of Software Systems and Cybersecurity, Monash University, Clayton, Victoria Australia

**Keywords:** Colorectal cancer screening, General practice, Primary care, Risk prediction tool, Implementation science

## Abstract

**Background:**

We developed a colorectal cancer risk prediction tool (‘CRISP’) to provide individualised risk-based advice for colorectal cancer screening. Using known environmental, behavioural, and familial risk factors, CRISP was designed to facilitate tailored screening advice to patients aged 50 to 74 years in general practice. In parallel to a randomised controlled trial of the CRISP tool, we developed and evaluated an evidence-based implementation strategy.

**Methods:**

Qualitative methods were used to explore the implementation of CRISP in general practice. Using one general practice in regional Victoria, Australia, as a ‘laboratory’, we tested ways to embed CRISP into routine clinical practice. General practitioners, nurses, and operations manager co-designed the implementation methods with researchers, focussing on existing practice processes that would be sustainable. Researchers interviewed the staff regularly to assess the successfulness of the strategies employed, and implementation methods were adapted throughout the study period in response to feedback from qualitative interviews.

The Consolidated Framework for Implementation Research (CFIR) underpinned the development of the interview guide and intervention strategy. Coding was inductive and themes were developed through consensus between the authors. Emerging themes were mapped onto the CFIR domains and a fidelity checklist was developed to ensure CRISP was being used as intended.

**Results:**

Between December 2016 and September 2019, 1 interviews were conducted, both face-to-face and via videoconferencing (Zoom). All interviews were transcribed verbatim and coded. Themes were mapped onto the following CFIR domains: (1) ‘characteristics of the intervention’: CRISP was valued but time consuming; (2) ‘inner setting’: the practice was open to changing systems; 3. ‘outer setting’: CRISP helped facilitate screening; (4) ‘individual characteristics’: the practice staff were adaptable and able to facilitate adoption of new clinical processes; and (5) ‘processes’: fidelity checking, and education was important.

**Conclusions:**

These results describe a novel method for exploring implementation strategies for a colorectal cancer risk prediction tool in the context of a parallel RCT testing clinical efficacy. The study identified successful and unsuccessful implementation strategies using an adaptive methodology over time. This method emphasised the importance of co-design input to make an intervention like CRISP sustainable for use in other practices and with other risk tools.

**Supplementary Information:**

The online version contains supplementary material available at 10.1186/s13012-022-01205-8.

Contributions to the literature

- This implementation study was conducted in parallel with an RCT which demonstrates an ability to conduct an implementation study in an isolated practice setting while testing the feasibility of the tool itself
- The CRISP tool which was being implemented was improved and designed with the feedback from practice nurses
- Implementation strategies were identified through trial and error in real time
- We have developed an evidence-based implementation strategy that can be used to prospectively implement tools into general practice

## Background

### The problem: the right screening for bowel cancer based on an individual’s risk

Colorectal cancer is the third leading cause of cancer mortality in Australia causing 5255 deaths in 2019 [1]. Screening with faecal occult blood testing can reduce mortality by detecting colorectal cancer early [2–5]. In Australia, the National Health and Medical Research Council (NHMRC) currently recommends biennial immunochemical faecal occult blood test (FIT) screening from 50 to 74 years for those at average risk of colorectal cancer, delivered through the National Bowel Cancer Screening Program (NBCSP) [6]. For those at moderately increased risk based on their family history of colorectal cancer, FIT-based screening is recommended from age 40 and colonoscopy screening every five years from age 50 [7]. Currently, many patients at average risk of colorectal cancer have colonoscopy screening instead of FIT screening, contrary to national clinical guidelines, while many patients at increased risk of colorectal cancer are not having colonoscopies when they should [8, 9].

### Development and clinical testing of CRISP

**Fig. 1:**
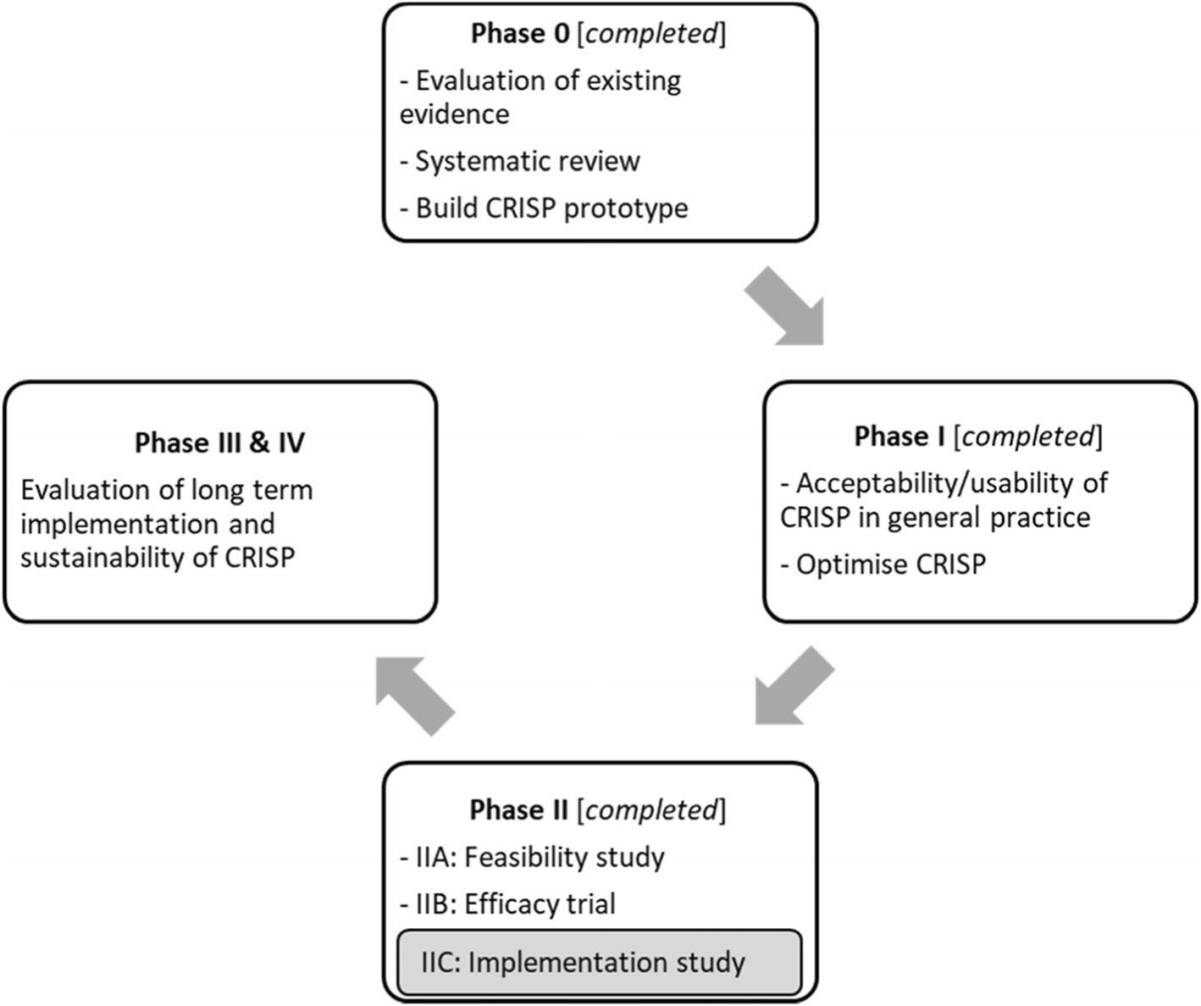
CRISP program of research based on the NHMRC guidelines for developing and evaluating complex interventions [11, 12]

In 2012, we developed and validated an Australian colorectal cancer risk prediction model which included several lifestyle factors, family history, medications and screening history [10] (Fig. 1) with the aim to incorporate it into a colorectal cancer risk prediction tool (‘CRISP’). The aim of CRISP was to improve risk-appropriate screening in general practice [13]. We conducted a systematic review of cancer risk tools in general practice [14], followed by a phase I study using simulated consultations to optimise the design of CRISP. The simulation study [11] also explored the context of colorectal cancer risk assessment and screening and collected preliminary data about the barriers and facilitators of using CRISP.

We conducted a Phase IIA/feasibility study testing the proposed methods for a randomised controlled trial (RCT) [15]. As a result of the feasibility study, we identified that practice nurses were best placed to use CRISP. Based on the results of the phase I and phase IIA studies, we conducted an efficacy RCT involving 732 patients in general practice, the results of which have recently been submitted for publication [13]. The trial demonstrated a 21% increase in risk-appropriate screening in those who were due some form of colorectal cancer screening. In parallel with the RCT, we sought to develop and evaluate approaches to implement the CRISP tool in practice, in a sustainable and scalable way. For this study, we used the Consolidated Framework for Implementation Research (CFIR) to underpin our data collection and analysis [16]. The implementation study would be conducted in a regional clinic in Victoria, Australia, that was not involved in the RCT, as to isolate the implementation and allow us to observe the methods independently to the trial.

#### The CRISP tool

The CRISP tool is described in detail within the efficacy trial protocol [13] and the tool itself can be accessed at: https://crisp.org.au/crisp-clinic.

### What is known and the research gap

Systematic reviews by the Cochrane Effective Practice and Organisation of Care group report several implementation strategies that have been effective in successfully translating health interventions into clinical practice [17]. Evidence-based interventions include providing educational materials for end users, training, educational outreach, support from local opinion leaders, audit and feedback, computerised reminder systems, and tailored strategies [17]. A systematic review conducted by our research group [14] reported that cancer risk tools were more likely to be successful if patients initiated them [18], if a member of the clinical team was given the task of completing them [17], if they included health promotion material [19], and/or if the tool provided evidence-based decision support when used in practice [20]. There are currently no detailed guidelines for implementing cancer risk assessment tools into clinical practice.

## Methods

### Research aims

In this qualitative study, we aimed to evaluate implementation methods to support the adoption of CRISP in a ‘real world’ clinical practice setting (Phase IIC). Specifically, we aimed to

- To develop an evidence-based implementation intervention capable of supporting the adoption of CRISP in general practice, in a sustainable way,
- To determine methods for scaling-up the implementation of CRISP into general practices in Australia, and
- To develop an approach to implement risk assessment tools for other cancers into general practice.

### Theoretical approach

This qualitative study used semi-structured focus groups and individual interviews of practice staff including receptionists, practice managers, nurses, and general practitioners. A constructivist paradigm was used as participants construct their understanding through experience [21], using interviews to explore their reflection on their experiences when using and implementing CRISP into their clinical practice. For this study, a semi-structured interview guide was developed based on the CFIR [22] (Table 1). See Supplementary file A for interview guide.

**Table 1:** Overview of the Consolidated Framework for Implementation Research (CFIR). The CFIR provides constructs that have been associated with effective implementation [22]

***Characteristics of intervention***	***Inner setting***	***Outer setting***	***Individuals involved***	***Implementation process***
- Intervention source - Evidence strength and quality - Relative advantage - Adaptability - Trialability - Complexity - Design quality - Cost	- Structural characteristics - Networks and communications - Culture - Implementation climate	- Patient needs and resources -Cosmopolitanism - Peer pressure - External policies and incentives	- Knowledge and beliefs about the intervention - Self-efficacy - Individual stage of change - Individual identification with organisation - Other personal attributes	- Planning - Engaging - Executing - Reflecting and evaluating

The CFIR outlines factors that influence implementation including the following: (1) *characteristics* of the intervention (intervention source, evidentiary support, relative advantage, adaptability, trialability, and complexity); (2) *the inner setting* (structural characteristics, networks and communications, culture, climate, readiness for implementation); (3) *the outer setting* (patient needs and resources, organisational connectedness (‘cosmopolitanism’), peer pressure, external policy and incentives); (4) *the characteristics of individuals* involved (knowledge and beliefs, self-efficacy, stage of change, identification with organisation, etc.), and (5) *the process* of implementation (planning, engaging, executing, reflecting, evaluating) [23]. The CFIR has been shown to be an effective framework for qualitative analysis, facilitating the evaluation of intervention implementation methods in a healthcare context [24, 25].

### Recruitment and consent

Researchers JM and JE had an existing research relationship with a rural Victorian general practice that expressed interest in participating in this study. It is a teaching practice that is affiliated with the University of Melbourne. Crucially, the practice nurses at this site had enough capacity to be involved. All practice staff, including nine general practitioners (‘GPs’), including six vocationally registered GPs and three registrars, and four practice nurses, provided written consent to participate in this study.

We obtained ethics approval through the University of Melbourne’s Medicine and Dentistry Human Ethics Sub-Committee (application ID: 1648457).

### Participant tasks

#### Planning and engaging

In 2016 and early 2017, JE, SS, and JM held two preliminary meetings with the whole practice team, including the nine general practitioners and four practice nurses, where they explained the implementation study. In the first meeting, the CRISP tool was demonstrated in detail as well as the rationale for using it in general practice. At the second meeting, it was agreed that the practice nurses would be the primary users of CRISP since they were enthusiastic, felt it would fit in well with the practice, and was consistent with our previous research results [15]. The model of implementation was that nurses would conduct the CRISP consultation with the patient, and afterwards the patient would attend their GP who would respond to any screening recommendations in the CRISP report.

These meetings included GPs, practice nurses, the practice manager, operations manager, and head receptionist. The discussions included detailing the clinical workflow and exploring how CRISP might be incorporated into existing appointments at the clinic or alternatively, the practice could contact eligible people and ask them to attend the clinic for a CRISP consultation. The co-design method involved discussions and decisions about how to introduce CRISP based on what the clinic staff wanted, felt was possible, and was common to other general practices.

#### Executing

##### Data collection

Since the practice did not start using CRISP until a year after the preliminary meetings (September 2018), researchers SM and JM gave a refresher training session to the GPs and practice nurses the day the nurses started using CRISP, to ensure they still understood their role and how CRISP worked. This included explaining how the clinical recommendations and risks were generated, and how to interpret outputs so that the GPs could manage the advice provided by CRISP. All CRISP consultations were conducted face-to-face by practice nurses.

**Fig. 2:**
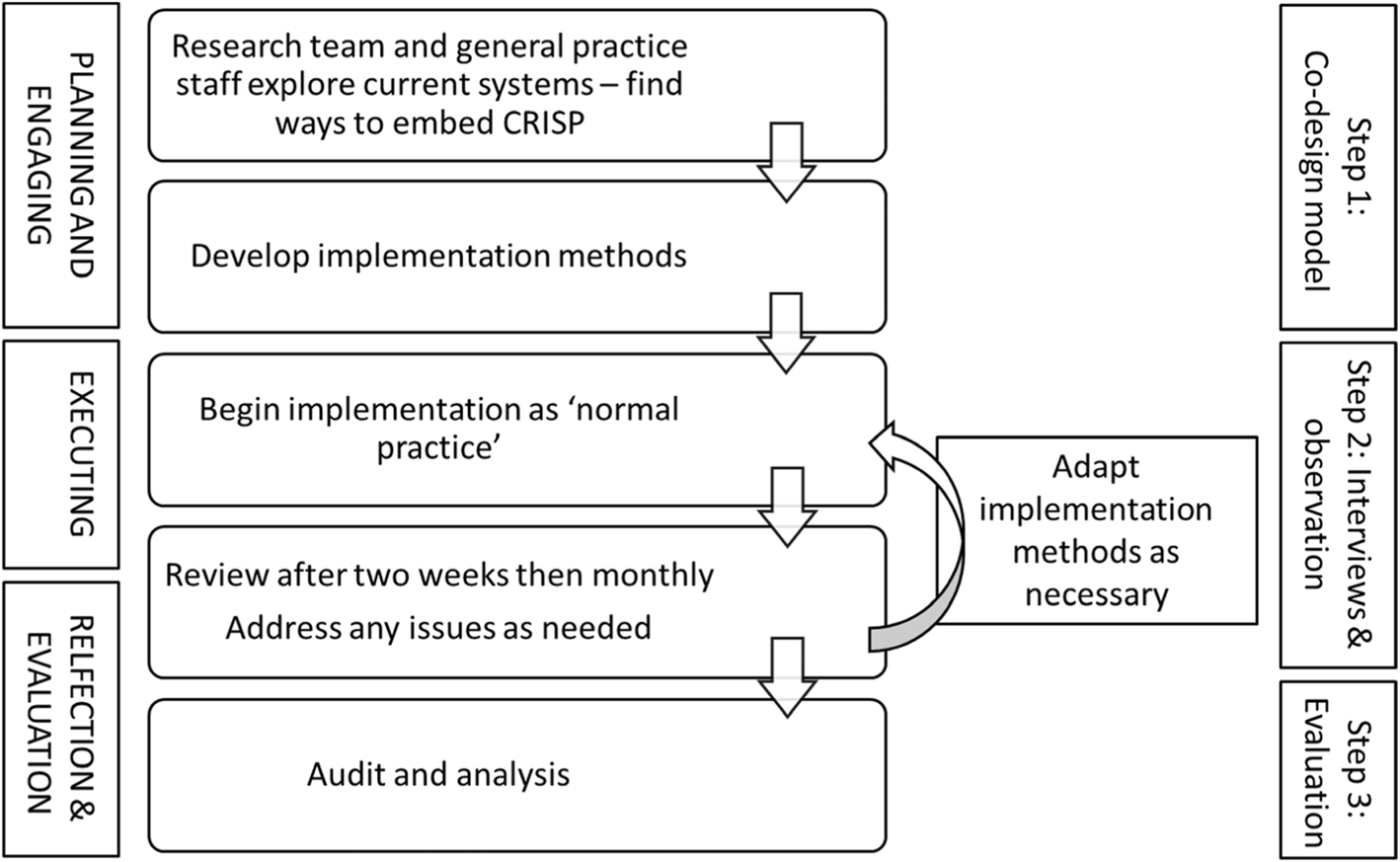
Overview of the methods used in the CRISP implementation study

From September 2018 to September 2019, the practice staff used CRISP and SM and JM interviewed the practice nurses throughout duration of it being used. The research team also spoke with staff over the phone and by email, troubleshooting any problems that occurred and checking-in with the staff about how the implementation was progressing. The interviews were conducted in the clinic at regular intervals with additional Zoom interviews. Interviews explored if, and how, CRISP was being used, which aspects were working well, and how existing approaches could be modified to increase uptake of the tool. Strategies were modified if the nurses found approaches to be ineffective, or if methods could be altered to increase use of the tool (Fig. 2).

Formal group interviews were conducted only with the practice nurses. During group interviews, field notes were taken by SM while JM facilitated. Interviews were audio-recorded and transcribed verbatim. Interviews were conducted in groups, pairs, and at times individually to avoid any potential influence arising from group dynamics. The interviews explored the five domains of CFIR to determine where the implementation strategies were successful or unsuccessful, and how the approaches could be adapted. Interview transcripts were not returned to participants.

The other practice staff including GPs and the practice manager were involved in the study but only gave brief updates about the CRISP in their monthly staff meetings and spoke to research staff about using the tool upon us visiting, only informal data was collected about these interactions.

#### Reflecting and evaluating

Researchers met regularly to review the interview transcripts, and discuss the data, emerging themes, and changes suggested by the nurses. Adaptations to the implementation methods were driven by interview data and additional feedback from the practice manager and GPs. Implementation methods were adapted only if the research team and practice staff agreed unanimously. This happened iteratively, as necessary throughout the study period.

To ensure CRISP was being used as intended, the researchers developed a fidelity checklist (Table 2) which was used during training, and later as a self-auditing tool by the nurses during CRISP consultations with their patients.

**Table 2:**
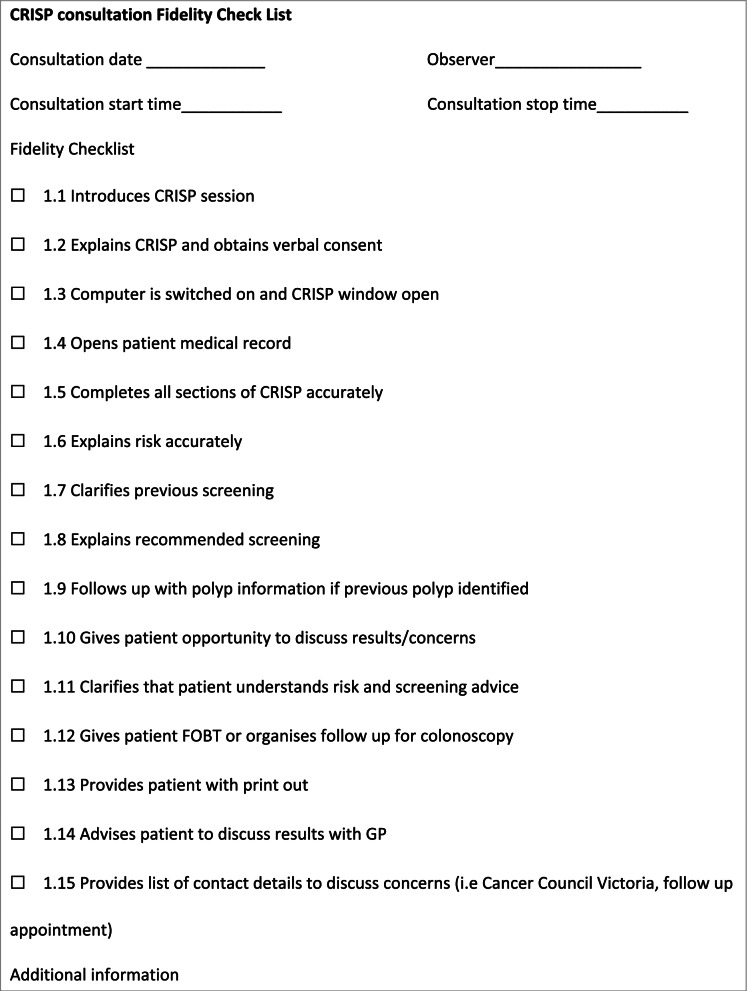
Fidelity checklist used to ensure CRISP quality delivery

#### Analysis

Qualitative analysis was managed using NVivo 12 [26]. Complete coding was employed by SM. After first-level coding, codes were grouped into themes. Themes were then mapped onto the constructs from the CFIR (15): characteristics of intervention, outer setting, inner setting, and characteristics of individuals and process (Fig. 2). To enhance interpretive rigour, at each level of coding, meetings with all researchers were held to discuss the coding and grouping of codes into themes and CFIR mapping.

### Participant incentives

The clinic was reimbursed $AUD 1000 for their involvement and time in the study. The practice nurses and the practice manager (JR, JK, GH, AC, and JW) were invited to be co-authors on all research outputs from the study, including this publication [27].

## Results

### Sample

A total of 13 interviews were conducted by SM, JM, and the practice nurses JR, GH, AC, and JW from September 2018 to September 2019. Interviews ranged from 45 to 75 min. Of the 13 interviews, 1 was conducted over Zoom, and 2 one-on-one interviews were conducted over the phone. All the nurses participated in the study for the entire study period except when on annual leave.

### Implementation strategies

Strategies to implement CRISP into practice are briefly covered in Table 3.

**Table 3:** A list of implementation strategies

The implementation strategies that were co-designed with practice nurses and other practice staff included: • nurse training that encompassed how to use the CRISP tool, how to explain the risk output, and how to inform the doctors of the result; • educational material designed for nurses, helping them explain CRISP results to the patients; • provision of a sample National Bowel Cancer Screening FIT kit for the nurses to demonstrate how to do a test with patients; • improved and more accurate instructions for patients on how to do a FIT test; • a fidelity checklist for research staff to verify that CRISP was being used correctly, and to help the nurses self-audit (Fig. 1); • training sessions for the doctors to ensure they understood the clinical implications of the CRISP output for their patients; • the incorporation of CRISP into preventive health consultations including chronic disease management plan consultations; • alerts in the patient management system, prompting the nurses to perform a CRISP assessment for eligible patients; • engagement with local pathology providers to explore barriers to FOBT kit availability outside the NBCSP; • engagement with local colonoscopic services to determine the availability of their services; and • identifying a ‘champion’ in the practice to drive the implementation.	

The practice staff suggested methods for introducing the CRISP into practice. The most popular method was to use CRISP during preventive health checks and chronic disease management consultations. The nurses often used these consultations to check on preventive health care such as cancer screening and found CRISP a useful tool to include in those sessions. A senior nurse accepted the responsibility of practice champion to lead the implementation of the CRISP. This senior nurse was also responsible for bowel screening in the practice, and so CRISP was within her established job domain. We discussed developing an alert to remind nurses that a patient booked for an appointment was eligible for a CRISP consultation, and we placed notices in the waiting room to inform patients about our research.

Strategies for implementing CRISP included how it could be incorporated into current consultations using Medicare Benefits Schedule items, i.e. medical services that can be charged to the MBS, Australia’s national healthcare scheme. Opportunistic and targeted methods were considered so that people eligible for colorectal screening could have a CRISP consultation when they attended the clinic for reasons other than a screening consultation.

To increase opportunistic and targeted ways to book an appointment for a CRISP consultation, practice nurses described and adapted methods to identify patients who were eligible for colorectal cancer screening. The nurses completed the CRISP tool with patients, printed out the results, provided the tool’s output, and discussed the screening recommendations prior to the patient seeing their GP. The patients followed up the CRISP results with their GP who made the final clinical decision about how, or if, the patient should be screened for colorectal cancer.

We met with the pathology providers and ascertained that FIT kits were available. However, we found that the FIT usage instructions in the clinic were outdated and not relevant to the new immunochemical FIT kits. We also discussed the accessibility of colonoscopies and found that they were not always readily available, sometimes resulting in a prolonged waiting period.

Many ideas to integrate CRISP tool with the practice’s Electronic Medical Record (EMR) system were discussed, including auto-populating data from the patient’s EMR and auto-populating risk scores back into a patient’s EMR, and having the tool accessible within the EMR. These were out of our budget and time frame, and consequently we were unable to pursue this work.

### Evaluation

We evaluated implementation strategies using interviews that adhered to the CFIR framework.

The results are described using the five CFIR domains to explain the emergent themes in the context of implementation barriers and facilitators (see Table 4).

**Table 4:** A summary of results of themes from interviews with; practice staff, including GPs and practice nurses mapped onto the Consolidated Framework for Implementation Research

*Characteristics of intervention*	*Inner setting*	*Outer setting*	*Individuals involved*	*Implementation process*
-CRISP was a valuable intervention and prompted them to discuss bowel cancer screening -As CRISP is a website, the nurses had trouble using the desktop shortcuts after the tool was updated -It was suggested that CRISP should be embedded within the electronic medical records - CRISP took time to complete and auto-populating fields from the electronic medical records would save time	- The general practice where CRISP was implemented changed a lot over the duration of the study - During flu season it was hard for practice nurses to find time to use CRISP -Nurses identified opportunities to use CRISP i.e. during cervical screening appointments -The clinic’s billing system changed, and some patients had to pay out of pocket	- CRISP encouraged participation in the National Bowel Cancer Screening Program - As there are long waiting times for colonoscopic screening in the public system, CRISP decreased the need for unnecessary ones - CRISP increased risk appropriate screening, so more the right people used the right screening methods	- The nurses were unaware of the risk factors for colorectal cancer that were presented in CRISP, so ongoing training was essential for its appropriate use - GPs were pressed for time and felt overwhelmed by having to discuss the CRISP recommendations with their patients who often presented with multiple health concerns	- The clinic is a teaching clinic and they were incredibly flexible and open to change which may not be the same for other clinics - The nurses were comfortable using the Fidelity Checklist, presented in Table 2, with their patients during consultations - CRISP was well received by the practice and patients during consultations

#### Characteristics of the intervention (CRISP)

Interviews provided an opportunity for practice nurses to discuss how the CRISP tool worked as part of their clinical practice and suggest ways to improve the tool.

The practice staff recognised CRISP as a valuable intervention that could improve their clinical practice. Specifically, the practice nurses liked that CRISP prompted them to talk about bowel cancer screening easily, but also helped facilitate discussions about other healthy lifestyle changes their patients could make (Quotation 1a and 1b).

1a “I think the tool itself is really good because it also helps us to focus on their diet and lifestyle, and it’s making people think more about proactive help.” (Practice nurse 1)

1b “Well it’s sort of like quit smoking, if we ask the question, we’re not necessarily asking them to quit smoking, but it’s raising awareness to their health issues. It’s the same with Pap smears and testicular screening. It’s just raising awareness. I think more and more people are becoming more educated about their health.” (Practice nurse 1)

In practice, CRISP is hosted on an external website, and to make it easier to access, the practice staff created a shortcut to the website on their desktop. However, when CRISP was updated on the website, the shortcut had to be manually updated which occasionally caused problems (Quotation 1c).

1c “The tool crashed on the GP as he had an older version [of the shortcut] on his desktop.” (Practice nurse 4)

To overcome this, the nurses suggested embedding CRISP into the patient EMR software as they had with other risk calculators (Quotation 1d). They also felt this would encourage them to use it more regularly as well.

1d “Just wondered if there’s some way of putting it into the EMR software so you could actually remember it like the geriatric depression thing…and things that you can just take down” (Practice nurse 3)

CRISP sessions also took a non-trivial amount of time to complete in the context of consultations which often required other clinical activities. The nurses identified opportunities to save time including auto-populating patient details from the practice’s EMR system (Quotation 1e).

1e “So, we were talking about how having the tool autofill some things. I know it’s been possible with us in the other program we were using so maybe we can try that? It would save a few minutes.” (Practice nurse 4)

#### Inner setting

The general practice was continually adjusting their priorities which impacted the use of CRISP. Despite the tool being designed to take about five minutes to complete, competing demands on practice staff’s time limited their capacity to use CRISP regularly (Quotations 2a, 2b). This was especially obvious during ‘flu vaccination season, during which vaccination consultation was prioritised over a CRISP consultation. Practice nurses did not have shifts covered when they went on annual leave, so at times the practice was not all full capacity, adding further workload pressure.

2a “...not gone off our radar at all or enthusiasm, it’s simply not been able to fit this in.” (Practice nurse 3)

2b “There could have been people we missed out on, if I was travelling well for time then I could use the CRISP tool with the patients but no I didn’t catch everyone, sometimes it is impossible to fit something extra into a consultation.” (Practice nurse 2)

Practice nurses were constantly thinking of ways to identify opportunities to use CRISP during patient visits to overcome the time barrier (Quotation 2c and 2d).

2c “Cervical screening because my nurse team usually have 30 minutes for cervical screening, usually. That’s probably been an opportunity where we really have been able to do it at the CRISP tool.” (Practice nurse 2)

2d “The care plans [chronic disease management plans], you know 30 minutes is very tight if you know the client well and the paperwork is fairly well organised, you could possibly fit it in there.” (Practice nurse 4)

Throughout the duration of the project, the clinic experienced substantial internal changes that affected their use of CRISP. The clinic transitioned from being a fully bulk-billing clinic to charging patients out-of-pocket fees for many services (Quotation 2e and 2f). While preventative health checks continued to be bulk-billed, the billing change resulted in it not being used as frequently as it could have been.

2e “[We have a] new billing system - some patients will be charged an out-of-pocket fee of $20” (Practice nurse 1)

2f “Patients who aren’t on a care plan must pay out of pocket for their visit to [the practice] now, they may be hard to recruit” (Practice nurse 1)

Although the practice faced many changes, the general culture of the staff was positive - they remained flexible and agile, and continued to be open to change (Quotation 2g).

2g “All four [of the nurses] have been championing this... They’ve been absolutely on board with it” (Practice nurse 2)

#### Outer setting

CRISP has been designed to increase risk appropriate screening, and this includes encouraging average risk patients to screen with the National Bowel Cancer Screening FIT kit rather than undergo unnecessary colonoscopies. The nurses recognised this was a benefit to using the tool as it not only provided individualised risk but also methods for communicating recommended screening advice (Quotation3a). Also, nurses were aware of the long waiting periods to access colonoscopies through the Australian public healthcare system (Quotation 3b). They understood that CRISP’s ability to steer average-risk patents towards FIT also had the potential to reduce the pressure on the public healthcare system by preventing unnecessary colonoscopies.

3a “I find it helpful in terms of trying to dial people away from colonoscopies because we have a lot who are captured by specialists who… they’ve had a colonoscopy and are immediately booked in for another one.” (Practice nurse 3)

3b “They want to know are they iron deficient, are they anaemic, and that’s how they are prioritising so the referral’s going to enter the public system and face a lengthy wait and if you’re raising with the patient, “based on your history we think colonoscopy is the way to go but you may have to wait 9 months to a year for an interval colonoscopy” (Practice nurse 3)

The CRISP tool and discussions about colorectal cancer screening prompted patients to participate in the National Bowel Cancer Screening program ; (Quotation 3c).

3c “… people throw the NBCSP kit in the bin, so I do think this will be useful once we start approaching more people and people get used to being asked questions about it…” (Practice nurse 1)

Practice nurses also wanted NBCSP FIT kits to help explain their use to patients and further promote colorectal cancer screening uptake. Researchers SM and JM ordered sample kits from the NBCSP for each of the practice nurses (Quotation 3d).

3d “If I actually have a demonstration kit to show the patients how to use it I probably will but if I need to leave the consultation room to try and find one I might not use it or show them how to do it…this could be another barrier to implementation” (Practice nurse 2)

At the time of this study, the NBCSP had not been fully implemented as a biennial program. General practices were expected to use local pathology companies to provide FIT kits to patients who were not up to date with colorectal cancer screening. In discussions with the local pathology providers, we realised that the FIT kit instructions being offered by the practice nurses were written for the older guaiac-based test. That is, the instructions were not applicable to the immunochemical FIT kit being used. These instructions included dietary restrictions and a more complicated sample collection, which might have deterred patients from undertaking the test. The instructions for the immunochemical test were updated in the practice to eliminate this barrier to colorectal cancer screening.

#### Characteristics of individuals

Practice nurses were unaware that ‘risk factors’ in the CRISP tool included some factors that increased bowel cancer risk, but also others which reduced risk (Quotations 4a and 4b). Furthermore, there was some misunderstanding about which patients might not be suitable for CRISP due to additional risk factors such as inflammatory bowel disease. This highlighted the need for comprehensive information and training to ensure correct use of the tool. (Quotations 4c and 4d).

4a “Why is calcium a problem? I take calcium for my osteoporosis… I thought I better stop taking my calcium tablets if it will increase my risk.” (Practice nurse 2)

4b “So HRT increases your risk of breast cancer if you take it for a certain period of time and calcium has some risks as well. So that’s one thing that we clarified with you and also the more information when it comes to the analgesic stuff as well.” (Practice nurse 1)

4c “When you say NSAIDs, is Panadol an NSAID?” (Practice nurse 3)

4d “… if someone has had significant bowel disease, like ulcerative colitis and diverticulitis the tool shouldn’t be used for them, right? (Practice nurse) [Researcher response: “That’s actually a very good question. If someone has diverticulitis they can be included if it is not significant as it is very common].” (Practice nurse 3)

Nurses reported that there were barriers for GPs to discuss the CRISP recommendations when they received the printed CRISP report. They felt overwhelmed if their patients presented with significant/multiple health issues, and a discussion of bowel cancer screening was also expected (Quotation 4e and 4f). Time pressures felt by GPs prohibited them from fully embracing CRISP (Quotations 4g).

4e “I think we have to give them a bit of time to get their heads around this. One of the GPs who was overwhelmed coped two patients who had a history of polyps from me.” (Practice nurse 2)

4f “Because the GPs must dig in the patient’s records, they don’t feel they can manage this. Patients are booked for one item per consult, the GPs have said that they want the patients to come back.” (Practice nurse 2)

4g “…the GPs felt overwhelmed talking to the patients about the tool as they have very limited time to do so” (Practice nurse 2)

#### Process

The overall culture of the practice meant that practice staff were open to changing and adapting to the implementation of CRISP. As a teaching clinic, practice staff were familiar with exploring new methods. Nurses were motivated and enthusiastic but recognised that this might not be the case in every clinic (Quotation 5a). They were open to introducing the tool into existing processes and were very forthcoming with new ways to incorporate CRISP into their clinical workflow. This included, for example, flagging specific patients aged 50 to 74 years old who were eligible for screening, and incorporating CRISP into chronic disease patients’ care plan appointments, which are typically longer than other consultations (Quotation 5b).

The practice nurses were comfortable using the fidelity checklist alongside CRISP with their patients, and confident the tool was being used as it was intended (Quotation 5c).

Overall, the practice nurses thought that CRISP was easy to use, and they reported that patients enjoyed working through it because it raised awareness. (Quotation 5d and 5e).

5a “If you look at a lot of things that happen in our clinics, I think we’re probably fairly motivated here. Not that other nurses aren’t but it depends on what they’re doing with the… the quicker and the … simpler you make it, the easier it will be to continue to apply in the longer-term basis.” (Practice nurse 2)

5b “But you’re quite right, because we flag things on our care plan, particularly the breast screening, cervical screening, FIT, that is the time to flag and I thought to myself, I rebook for this, I give you the phone number for that, I’ve got time I’ll even do it on the phone in my room immediately.” (Practice nurse 4)

5c “We usually go through a bit of a fidelity checklist which I know we’ve done that with you before and it seems like that was all… everything was being used in the right way.” (Practice nurse 1)

5d “Mine have been fairly straight forward if I’m lucky.” (Practice nurse 4)

5e “I found that the patients I asked were very keen and liked going through the tool, it’s great at bringing awareness tool and gets people talking about their risk” (Practice nurse 3)

## Discussion

CRISP is a personalised colorectal cancer risk prediction tool to increase risk appropriate screening [13]. This study described methods to implement CRISP into general practice. We demonstrated how CRISP might be integrated into practice using evidence-based methods including ongoing training, identifying a practice champion, and using existing practice management systems to integrate CRISP into day-to-day clinical practice. We found that regular engagement with clinical staff, co-design, and ongoing iterative changes helped adapt implementation methods and adjust to changes within the general practice over time. Understanding the context of the clinic systems, culture, funding model and workflow was also essential. We also discovered the importance of understanding how CRISP would be affected by, and impact, the local external health system—in this case, endoscopy services and pathology providers.

### Proposed implementation strategy

Overall, clinic staff responded positively to the tool. Training the staff to use CRISP was beneficial. This helped them understand how personalised colorectal cancer risk estimates and could facilitate appropriate screening. This included understanding why the use of an FIT kit by patients at average risk might prevent unnecessary colonoscopies [28]. CRISP facilitated discussions with patients about their general health, including managing weight, smoking, and exercise—all of which are risk factors for colorectal cancer and many other health problems. CRISP also provided education for nurses on preventive colorectal cancer risk factors including aspirin, hormone replacement therapy, and calcium which they had not known previously. Ongoing interviews and fidelity checking provided the opportunity to ensure CRISP was used as it was intended and provided practice staff a chance to co-design the tool, making suggestions to improve its ease of use. Their suggestions included embedding the tool into the practice’s EMR software to make it more accessible, acting as a reminder for them to use it in consultations, auto-populating patient data from the EMR into CRISP, and automatically integrating resultant risk information back into the EMR. However, there remain significant and non-trivial challenges in the integration of external tools within commercial EMR systems.

### The CFIR framework

The CFIR framework was utilised to qualitatively evaluate our approaches to implementation, from which we determined that CRISP was well understood, liked by patients and staff, and utilised appropriately. We also determined that CRISP required training and had to adapt to changes in the clinic structure. Time and conflicting priorities were a barrier, and at times CRISP was overlooked in favour of more pressing issues such as flu vaccination and patient-driven needs. The comprehensiveness of CFIR and the ongoing engagement with the practice staff was beneficial in evaluating all aspects of CRISP implementation.

Many colorectal cancer risk prediction tools have been developed and validated internationally [29–31] and, despite many suggesting implementation is the next important step after a tool has been developed [32, 33], none have outlined implementation strategies that may have been employed. Nonetheless, we built our methods based on the limited evidence from our initial systematic review of cancer risk assessment tools in primary care. We included health promotion, a dedicated clinician (the practice nurse), and decision support, and we tested it in a real-world setting.

### Limitations

There were several limitations to this study. Firstly, general practice is an ever-changing environment, and the clinic where we implemented CRISP was no different. In Australia, there is a shortage of rural GPs; they move frequently and work in these areas for a specified amount of time [34]. Although we did not record these data in our interviews, we observed that this was true of the clinic in which we conducted our study. During our time in the clinic, the billing system changed from being a fully bulk-billing (essentially no cost to patients) clinic to a mixed-billing clinic whereby patients would be charged a $20 out-of-pocket fee to see a GP. These changes can impact the patient population, staff load, and established systems in clinics, and affect their priorities. These factors can negatively impact an established implementation method.

CRISP was only implemented into one general practice and due to the diversity of clinics, the implementation framework this project generated may not be fully scalable to another clinic. We attempted to minimise this by using implementation methods that are common to many general practices; however, each practice has its own inner setting characteristics that can affect the success of implementation. The evolving nature of general practice poses a challenge to the long-term implementation of tools such as CRISP.

The final limitation was the absence of quantitative data. We did not intend to recruit a sample large enough to measure any changes over time in response to using CRISP. This would be an important addition to a larger implementation study. Logging of quantitative CRISP session data (time elapsed, screen navigation flows, etc.) was not in scope for this research but may be useful in the future to understand and assess uptake of risk prediction tools.

## Conclusions

This research has identified a method for implementing CRISP in general practice by applying co-design in situ within a clinical setting. Our learnings have significant value—while the findings presented here involve a risk assessment and screening tool for colorectal cancer, a leading cause of cancer mortality, they may also be applicable to the implementation of tools for other diseases. The methodology we used for evaluating implementation methods in general practice demonstrated the necessity for ongoing training, fidelity checking, and understanding the context of the clinic and related health services external to the clinic. The use of co-design and engagement over time with clinic staff was beneficial for establishing, reviewing, and adapting implementation methods for the CRISP tool. Although general practices vary, the methodology used to implement CRISP could be used across many general practices, used on a larger scale and supported by quantitative evaluation. We suggest this method could be used on a larger scale and supported by quantitative evaluation.

## Supplementary Information

**Additional file 1: Supplementary file A.** Interview guide.

**Additional file 2: Supplementary file B.** TIDIeR checklist for the intervention.

## Data Availability

Non-digital data was stored in a locked cabinet on the 10th Floor of the Victorian Comprehensive Cancer Centre. SM, JM, and JE will be responsible for the short and long-term storage. Research data and primary materials will be stored for a minimum of 5 years after the last publication, or public release, arising from the research as per University of Melbourne Code 2.1. (University Code§2.1).

## Abbreviations

CRISP: Colorectal cancer RISk Prediction tool
CFIR: Consolidated Framework for Implementation Research
FIT: Immunochemical faecal occult blood test
GP: General practitioner
NBCSP: National Bowel Cancer Screening Program
NHMRC: National Health and Medical Research Council
