# Supplementary material for: Exploring a novel method for optimising the implementation of a colorectal cancer risk prediction tool into primary care: a qualitative study
Source: Implement Sci. 2022 May 12;17:31. doi: 10.1186/s13012-022-01205-8 (PMC9097304; doi:10.1186/s13012-022-01205-8)
Supplement: Supplementary file 1 — Additional file 1: Supplementary file A. Interview guide. [file 13012_2022_1205_MOESM1_ESM.docx]

Supplementary File A. Interview Guide

**CRISP IIC – INTERVIEW SCHEDULE**

**Consolidated Framework for Implementation Research (CFIR) questions** [**http://cfirwiki.net/guide/app/index.html#/guide_select**](http://cfirwiki.net/guide/app/index.html%23/guide_select)

***Commence interview***

**INTERVIEW QUESTION GUIDE WITH SAMPLE QUESTIONS.**

[The interview will include the following questions, prompts and probes but will not be restricted to these specific questions and not necessarily be worded in exactly the same way given that the method is more of an action design and will require some flexibility.]

1. Prior to using CRISP, did/how did you assess risk of bowel cancer and screening with patients?

**CFIR -** How do people feel about current practices that are available related to the intervention?

*Potential probing questions:*

- ***CFIR*** *- Will the intervention replace or compliment a current program or process?*
- *NHMRC guidelines for bowel cancer screening? National Bowel Cancer Screening program?*
- *What prompts you to discuss bowel cancer screening with your patients?*

2. REACH

Since using CRISP, who would you use with it in your clinical practice?

**CFIR** - How will you or your colleagues communicate to the individuals that are served by your organization about the intervention?

How do you feel about the intervention being used in your setting?

*Potential probing questions:*

- ***CFIR -*** *How confident do you think your colleagues feel about implementing the intervention?*
- ***CFIR -*** *How confident do you think your colleagues feel about using the intervention?*

**CFIR -** What steps have been taken to encourage individuals to commit to using the intervention?

*Potential probing questions:*

- *Notices in the waiting room*
- *Prompts on your computer*
- *Other implementation strategies that might have increased the awareness of CRISP*

3. EFFICACY

In your opinion, was CRISP effective?

**CFIR** - Do you think the intervention will be effective in your setting?

**CFIR** - How does the intervention compare to other alternatives that may have been considered or that you know about?

4. ADOPTION

Have you used CRISP to discuss bowel cancer risk and testing with a patient? If so, how did the

consultation go?

**CFIR** - Have you elicited information from participants regarding their experiences with the

intervention?

*Potential probing questions:*

o ***CFIR*** *- How confident are you that you will be able to use the intervention?*

o *How did you feel using CRISP?*

o *How confident were you in your ability to use CRISP? What made/would make you feel more*

*confident?*

5. IMPLEMENTATION

How did you find the strategies that we incorporated into the study to increase the use and ease of

use of CRISP?

*Potential probing questions:*

o ***CFIR*** *- What kinds of changes or alterations do you think you will need to make to the*

*intervention, so it will work effectively in your setting?*

o *Did you notice them? If yes, was that a positive or negative thing?*

o *Were there any methods that were never used? Prompt: check implementation strategies as*

*necessary*

o *Would you suggest anything else that might be used/developed to increase the*

*implementation of CRISP within the clinic system?*

o *Why/why not? (all above)*

6. MAINTENANCE

Did you use CRISP as much since I last spoke to you or less or about the same?

*Potential probing questions:*

o *Is it easy to maintain or difficult? Why/why not?*

o *Did you remember to use CRISP?*

o *Do you think that you and your colleagues will use CRISP in the future?*

o *Do you have any ideas about how CRISP might become more sustainable?*

o *Would you consider using a similar tool for other types of cancer screening?*

o *What might facilitate you using it as a regular tool?*

7. Is there anything else that you would like to say about using CRISP?

8. Is there anything else you would like to say about bowel cancer screening and testing?

• Ok, thank you. That’s all of my questions for now. We will be doing other interviews in a few

weeks to a month from now.

• Set up next interview
